# Young key populations and HIV: a special emphasis and consideration in the new WHO Consolidated Guidelines on HIV Prevention, Diagnosis, Treatment and Care for Key Populations

**DOI:** 10.7448/IAS.18.2.19438

**Published:** 2015-02-26

**Authors:** Rachel Baggaley, Alice Armstrong, Zoe Dodd, Ed Ngoksin, Anita Krug

**Affiliations:** 1 HIV Department, World Health Organization, Geneva, Switzerland; 2 International Network of People Who Use Drugs, Toronto, Canada; 3 Global Network of People Living with HIV, Cape Town, South Africa; 4 Youth Research Information Support Education (Youth RISE), Melbourne, Australia

WHO released its new *Consolidated Guidelines on HIV Prevention, Diagnosis, Treatment and Care for Key Populations* [1] at the International AIDS Conference in Melbourne in July 2014. This guidance addresses five key populations: men who have sex with men, people who inject drugs, people in prisons and other closed settings, sex workers and transgender people. For the first time in its work on key populations, WHO chose to specifically address adolescent and young key populations, considered specific adolescent issues relating to all recommendations and implementation considerations, highlighted case examples and discussed challenges and barriers to acceptable and effective service delivery. In addition, four technical briefs, developed by the Interagency Working Group of Key populations, on HIV and young men who have sex with men, young people who sell sex, young people who inject drugs and young transgender people have been included as annexes to the guidelines.

## High HIV risk: limited data

**Figure 1:**
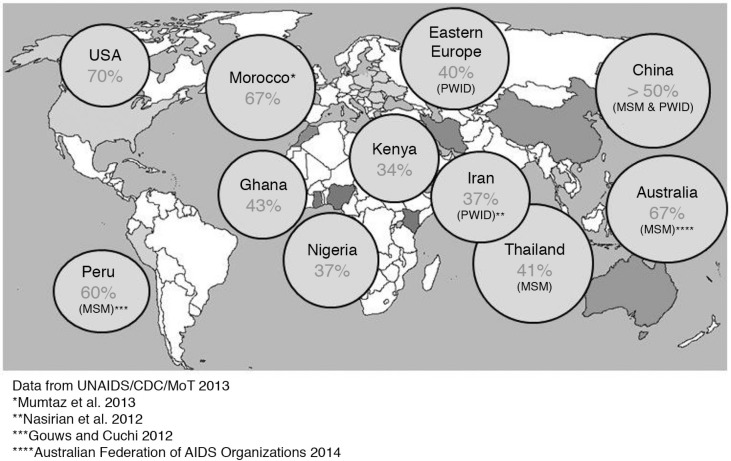
New infections attributable to key populations. From Ref. [3].

In all epidemic contexts, HIV incidence remains high or is increasing among key populations (Figure 1). Currently, there is a lack of global data pertaining to estimates of adolescent and young key populations, as well as their risks and needs. Where accurate surveillance data for young key populations are available, the HIV prevalence among these groups is often found to be significantly higher than that of the general youth population [3]. Available data are often not disaggregated by age, and those under 18 years are underrepresented in research. However, what we do know paints a stark picture.

According to the report of the Commission on AIDS in Asia, nearly all (95%) new HIV infections among young people in Asia occur in young key populations. In this region, however, over 90% of HIV resources for young people are focused on programming for “low-risk youth” [4]. Furthermore, studies consistently demonstrate that young key populations are even more vulnerable than older cohorts to sexually transmitted infections, including HIV and other sexual and reproductive health concerns [5–9].

Available data also suggest adolescent key populations are disproportionately affected by HIV in almost all settings [10]. For example, pooled data show significantly higher HIV prevalence and increasing rates of new HIV infections among adolescent men who have sex with men than among men of the same age in the general population [11,12]. Among adolescent males aged 13–19, in the United States, 92.8% of all diagnosed HIV infections were attributed to male-to-male sexual contact [13,14]. HIV infection rates ranging from 9 to 22% have also been reported in a variety of small, non-representative samples of adolescent transgender females [15,16]. Such reports are notable and significantly higher than the HIV prevalence reported in other adolescent study samples [9,17]. Adolescent transgender females with a history of selling sex may be more than four times as likely to be HIV-infected than their peers [18].

The age at which young people start to engage in behaviours that place them at higher risk of HIV is diverse and varies by country and context; however, evidence shows some begin high-risk behaviours during adolescence. In community consultations, most young people reported starting to inject drugs between 15 and 18 years [19]. In a study among 10–19 year olds living or working on the streets in four cities of Ukraine, 45% of those who reported injecting drugs said that they began doing so before they were 15 years old [20]. Behavioural surveillance indicates that in India 17% of female sex workers initiated selling sex before the age of 15 years, while those in Papua New Guinea reported a mean age of initiation of 17–19 years [21,22].

Although there are unique and diverse issues which contribute to the particular vulnerabilities of adolescent and young key populations, it is also important to recognize their strengths, capacities and resilience, and to recognize these in developing and supporting services and responses to their needs.

## Barriers to services: poor service provision

Young key populations are not adequately reached with appropriate and acceptable HIV prevention, treatment and care interventions and services. Many barriers limit their access to these essential services, or exclude them from using formal health services altogether. Notably, policy and legal barriers related to age of consent to accessing a range of health services including HIV testing and counselling, sexual and reproductive health, harm reduction, and other services provided specifically for key populations limit the ability of young individuals to exercise their right to independent decision-making and prevent them from accessing essential services. For example, in sub-Saharan Africa at least 33 countries have age-based criteria for consenting to HIV testing; 14 of which assert that only a person 18 years of age and above can consent to an HIV test [23].

Adolescents from key population groups are also often subject to significant levels of stigma, discrimination and violence. In many settings, laws that criminalize behaviours such as drug use, sex work and same-sex relationships further marginalize young people and perpetuate their social exclusion from their communities and essential support services. Fearing discrimination and possible legal consequences, many adolescents from key population groups are reluctant to attend HIV testing and treatment services. As such, they remain hidden from services and support networks and are often reluctant to disclose their HIV status to parents and family members in fear of revealing their identity or risk behaviour.

Additionally, most health services are not designed to care for, and address the needs of, adolescents and young people from key populations. Often services are delivered by staff who do not have experience or training in providing care and services for adolescents, and therefore may lack the sensitivity required to work with adolescent key populations. In other settings, services are simply not available, for example, for young transgenders. Available data indicate that young key populations may find services delivered through community and outreach-based programmes more acceptable than those provided in government facilities. This may be in part due to the impact of discriminatory policies including age restrictions, lack of confidentiality, mandatory registration and attitudes towards adolescent and young key populations within facility-based services [24].

## The new WHO guidelines

The new *Consolidated Guidelines on HIV Prevention, Diagnosis, Treatment and Care for Key Populations* have been developed in collaboration with key partners including community-based networks led by and/or for young key populations. They were based on reviews of available peer-reviewed published and grey literature (literature not available through the usual bibliographic databases, for example, programme and project reports), community consultations with young key populations and an extensive effort to collect case examples of good practices from programmes and organizations providing services to key populations. The case studies provide concrete practical examples of services or young key populations and highlight examples of their critical roles in developing and delivering these, including in youth-led advocacy, leadership and empowerment. They summarize the key issues facing key populations and underscore the importance of implementing a comprehensive package of evidence-based services and developing a national strategy to address their unique and diverse needs (Table 1).

**Table 1:** The comprehensive package of HIV prevention, treatment and care interventions and strategies for adults and adolescents as cited in the WHO key population guidelines

Essential health sector interventions	
1.	Comprehensive condom and lubricant programming.
2.	Harm reduction interventions^a^ for substance use (in particular needle and syringe programmes^b^ and opioid substitution therapy).
3.	Behavioural interventions.
4.	HIV testing and counselling.
5.	HIV treatment and care.
6.	Sexual and reproductive health interventions.^c^
7.	Prevention and management of co-infections and other co-morbidities, including viral hepatitis, tuberculosis and mental health conditions.
Essential strategies for an enabling environment	
1.	Supportive legislation, policy and financial commitment, including decriminalization of behaviours of key populations.
2.	Addressing stigma and discrimination.
3.	Community empowerment.
4.	Addressing violence against people from key populations.

a This package is essentially the same as the comprehensive package for HIV prevention, treatment and care for people who inject drugs that has been widely endorsed at the highest level [25,26]. For people who inject drugs, the harm reduction component of the package, and in particular the implementation of needle and syringe programmes and opioid substitution therapy, remains the first priority

b needle and syringe programmes are important for those people who inject drugs and also for transgender people who require sterile injecting equipment to safely inject hormones for gender affirmation. Other important areas include for tattooing, piercing and other forms of skin penetration, which are particularly relevant in prisons and other closed settings

c including contraception, diagnosis and treatment of sexually transmitted infections, cervical screening, etc.

From Ref. [1].

This comprehensive package recommends interventions and strategies relevant for adolescents and adults. The guidelines bring together relevant existing adolescent recommendations such as on HIV testing and counselling as well as provide additional specific adolescent considerations for overall recommendations. For example, in addressing legislative and policy barriers, additional adolescent considerations regarding age of consent barriers are specified.

Furthermore, the guidelines highlight that it is urgent for countries to review their legal policies, initiate the provision of services as well as improve services, include adolescent and young key populations in developing acceptable services and offer opportunities for their meaningful inclusion in defining their HIV and health service needs, developing effective services and participating in research. The resourcefulness and expertise of adolescents and young people is widely recognized, and their empowerment and inclusion in the design and delivery of research, services and interventions is promoted in many settings. In relation to HIV, much can be learned from listening to and involving young people regarding the strategies they already use in keeping themselves and their peers and partners safe, and in finding ways to more easily, safely and sustainably engage with health and other forms of care and support, despite the often considerable barriers and constraints.

Urgent attention must however be given – and practical ways of working within legally constrained settings sought – in order to provide services for young key populations and to prevent their continuing vulnerability to and risk of HIV infection, and to ensure equitable access to HIV testing, treatment and care. We hope that the new guidelines will catalyze better programming for adolescent and young key populations and legitimize their role in designing, developing and delivering them.
